# Determination of the Maximum Temperature in a Non-Uniform Hot Zone by Line-of-Site Absorption Spectroscopy with a Single Diode Laser

**DOI:** 10.3390/s18051608

**Published:** 2018-05-17

**Authors:** Vladimir V. Liger, Vladimir R. Mironenko, Yurii A. Kuritsyn, Mikhail A. Bolshov

**Affiliations:** 1 Institute for Spectroscopy, Russian Academy of Sciences, 5 Fizicheskaya Str., Troitsk, 108840 Moscow, Russia; liger@isan.troitsk.ru (V.V.L.); miron@isan.troitsk.ru (V.R.M.); kuritsyn@isan.troitsk.ru (Y.A.K.); 2 Chemistry Department, Analytical Chemistry Division, Lomonosov Moscow State University, 1-3 Leninskie Gory, 119991 Moscow, Russia

**Keywords:** absorption spectroscopy, TDLAS, non-uniform temperature distribution, data processing, correlation analysis

## Abstract

A new algorithm for the estimation of the maximum temperature in a non-uniform hot zone by a sensor based on absorption spectrometry with a diode laser is developed. The algorithm is based on the fitting of the absorption spectrum with a test molecule in a non-uniform zone by linear combination of two single temperature spectra simulated using spectroscopic databases. The proposed algorithm allows one to better estimate the maximum temperature of a non-uniform zone and can be useful if only the maximum temperature rather than a precise temperature profile is of primary interest. The efficiency and specificity of the algorithm are demonstrated in numerical experiments and experimentally proven using an optical cell with two sections. Temperatures and water vapor concentrations could be independently regulated in both sections. The best fitting was found using a correlation technique. A distributed feedback (DFB) diode laser in the spectral range around 1.343 µm was used in the experiments. Because of the significant differences between the temperature dependences of the experimental and theoretical absorption spectra in the temperature range 300–1200 K, a database was constructed using experimentally detected single temperature spectra. Using the developed algorithm the maximum temperature in the two-section cell was estimated with accuracy better than 30 K.

## 1. Introduction

Optical sensors based on tunable diode laser (DL) absorption spectroscopy (TDLAS sensors) are widely used for contactless diagnostics of the parameters of different hot zones, in particular of the combustion zones of mixing flows of a fuel and oxidant [1,2,3,4,5]. This technique provides remote, non-perturbing measurements of the parameters of a hot zone with time resolution in the micro- to millisecond range depending on the specific experimental conditions. The basic advantages of TDLAS sensors are: relatively simple construction, relatively low cost of the commercial components of the sensor, and the possibility to locate the sensitive part of a sensor away from the hot and/or harsh zone using optical fibers to deliver the probing laser beams to the testing zone. Last but not least, the demand on the qualification of an operator is not extremely high.

Diverse TDLAS sensors for typical molecular components in a harsh environment have been designed. In general, such sensors are based on the detection of the absorption spectra of a test molecule. The main parameters of a hot zone (temperature, total pressure, concentration of a test molecule) are usually inferred by the fitting of experimental spectra, and theoretical ones simulated using spectroscopic databases. The most popular and well developed sensors are based on H_2_O as a test molecule. For diagnostics of a combustion process, water vapor concentration is a key criterion for measuring process efficiency.

One of the serious drawbacks of a simple one-dimensional sensor is the measurement of a mean temperature, which is in fact an “effective” (*Т_eff_*) temperature along the laser beam line-of-sight (LOS) [6,7,8,9]. Information about hot zone parameters is inferred from the absorption line intensities formed in the test zone. At the same time, the probing beam can be absorbed in the LOS not only in the hot zone but also as it passes from diode laser to the detector. For H_2_O sensors two primary effects modify the line shapes of the transmitted probing beam: (1) absorption of the probing beam outside the test volume; and (2) intrinsic gradients of the hot zone parameters—temperature and absorbing species number density. Evidently, the fitting of such modified line shapes by theoretical line shapes constructed using spectroscopic databases HITRAN/HITEMP [10,11] with a single temperature and partial pressure of the absorbing species results in the inferring of “effective” parameters. Both effects mentioned above result in a decrease of the *Т_eff_* compared to the maximum temperature of the hot zone as the temperature of laboratory air is definitely lower and, besides, the temperature of the hot zone decreases from its maximum value to the windows or walls of a test chamber.

The influence of the first effect can be reduced in different ways. One can purge the path of LOS outside the test zone with an inert gas, thus reducing the absorption of the probing beam in the laboratory air. Selection of so called “hot” absorption lines with lower energy levels of the transitions above, say, 1000 cm^−1^ also significantly reduces the absorption in room temperature laboratory air. A more radical approach is the selection of another test molecule [12]. However, this has the drawback of missing the valuable information on water vapor concentration, which is essential for the estimation of the efficiency of combustion processes. In any case, the first effect can be significantly reduced, leaving the temperature gradient in the hot zone as the intrinsic problem of a one-dimension sensor and neither of the above modifications provides an adequate solution.

Tomography is the most adequate scheme for diagnostics of a non-uniform hot zone [13,14]. However, this approach dramatically complicates the optical and electronic schemes of a sensor and the data processing of the experimental results. Even more serious is the impossibility to probe a hot zone inside real propulsions and engines. Tomography works perfectly for the diagnostics of open flows, for instance, for engine exhausts.

Two conceptual approaches for the diagnostics of non-uniform zones using a single DL beam were proposed and experimentally tested in [7,14,15]. Both rely on simultaneous measurements of multiple absorption transitions with different temperature dependences to extract the temperature distribution information along the LOS and are named *profile fitting* and *temperature binning*. The first approach is based on a postulated (a priori) temperature distribution along the LOS [15]. The second strategy determines the temperature probability density function along the LOS using prescribed temperature bins [7,14,15]. The temperature-binning method is useful for cases where the temperature distribution of the flow fields cannot be represented by a simple profile or where an exact temperature distribution profile is not necessary for the application. In both cases more advanced and expensive DLs with much broader tuning range (up to 10–15 cm^−1^) should be used to probe the absorption lines with significantly different lower energy levels. Both algorithms are based on the measurements of the integral line intensities which presume that spectral lines are not overlapped. In real propulsions systems this assumption is not fulfilled.

The aim of the present paper is the development of a new approach for evaluation of the maximum temperature in a non-uniform hot zone. Contrary to abovementioned procedures—profile fitting and temperature binning—we propose to fit the experimental absorption spectra by linear combination of two single temperature spectra (STS) with the appropriate component weights. The best fitting is found in this case by a correlation technique. This approach can infer the temperature which best coincides with the maximum value within a non-uniform zone and may be useful if only maximum temperature, but not spatial distribution, is the goal of the measurements.

## 2. Idea of the New Approach

In real hot zones (engines, engine exhausts, propulsions and power stations, etc.) the temperature is distributed non-uniformly. Our approach can be useful if the maximum temperature of a non-uniform hot zone is important, but not an actual temperature distribution in the LOS. The maximum temperature in a combustion zone is of primary importance for the evaluation of the efficiency of the process and fuel consumption.

Our approach is based on a two-temperature model. A hot zone is composed of a portion with high temperature *Т_high_* and low temperature *Т_low_*. The second important idea of the new approach is the use of a correlation technique to find the best fitting of the experimental and simulated absorption spectra [16]. These simulated spectra for non-uniform LOS are constructed as the linear combination of the single temperature theoretical spectrum with two different temperatures *T*_1_ and *T*_2_ with the variable coefficients. The best fit provides both minimum and maximum temperatures *Т_low_* and *Т_high_* for two-zone LOS.

The absorption transitions of a water molecule with lower energy levels in 1500–2000 cm^−1^ range should be selected to increase the contribution of the hot part of the LOS in the resulting line profile of the transmitted probing laser beam. The so called “cold” spectral lines with lower energy levels in 200–500 cm^−1^ range should be also used for estimation of the part of the LOS with a temperature gradient from the maximum temperature to the cold outer periphery of a hot zone.

**Figure 1:**
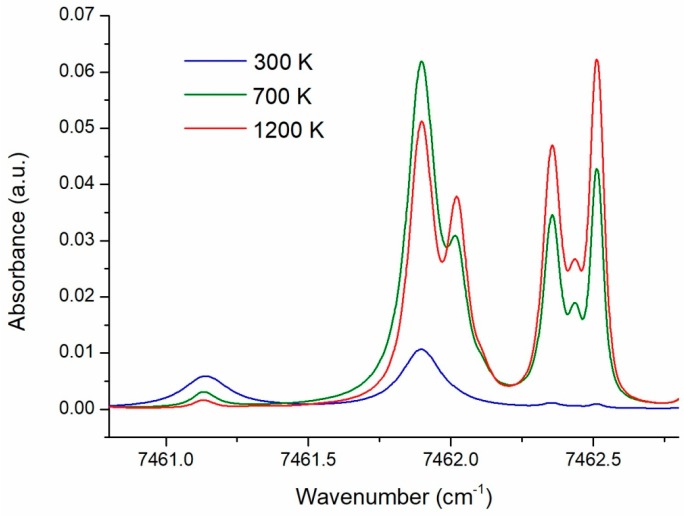
Three H_2_O absorption spectra in the atmosphere simulated using the HITRAN2016 database at: 300 K (blue), 700 K (green), and 1200 K (red). The total pressure was 1 atm, the pressure of H_2_O was 0.01 atm.

For the numerical and experimental investigations the lines in 7460–7463 cm^−1^ range were selected in this work. Both “hot” and “cold” lines are located in this range within an interval of about 2.5 cm^−1^, which can be scanned by a single distributed feedback (DFB) DL. The structure of the selected spectral range simulated using HITRAN2016 is shown in Figure 1. In this figure the spectra for three different temperatures are shown. For total pressure ≥1 atm the lines are overlapped, which makes it impractical (e.g., dramatically complicated) to define actual integral line intensities for evaluation of the temperature using the traditional algorithms.

## 3. Theoretical Background

In the case of thermodynamic equilibrium and uniform conditions, the temperature of a test zone is defined by the ratio of the integral intensities of two absorption lines with different lower energies. For the case of small absorption when the linear expansion of the Beer-Lambert law is valid, the measured absorption signal *Y*_ν_ at the wavenumber ν can be presented in the form:

(1) $$Y_{\nu }=\sum_{j}S_{j}(T)g_{j\nu }NL+b_{\nu }+\varepsilon_{\nu }=Z_{\nu }+b_{\nu }+\varepsilon_{\nu }$$

where *S_j_*(*T*) is the integral intensity of the line *j*, *g_j_*_ν_ is the line profile, *N* is the number density of the absorbing species, *L* is the length of the absorbing layer, *Z*_ν_ is the theoretical absorption spectrum simulated using a spectroscopic database, *b*_ν_ is the baseline (BL), and ε_ν_ is the residual (difference between the experimental and simulated spectra). The integral intensities are defined by fitting the experimental and theoretical spectra to minimize the residual. Expression (1) describes the absorption in a uniform medium with constant temperature *T* along the line-of-sight of the probing DL beam.

For a non-uniform temperature distribution this assumption is not valid. Our approach is based on the presentation of the non-uniform zone as the combination of a “cold” part with temperature *T*_1_ and “hot” part with temperature *T*_2_. For such a stepwise distribution, a theoretical absorption spectrum Z_ν_ can be presented in the form of a linear combination of the two spectra for two different temperatures *T*_1_ and *T*_2_:

*Z*_ν_ = *B*_1_*D*_ν_ (*T*_1_) + *B*_2_*D*_ν_ (*T*_2_), (2)

where *D*_ν_(*T*_1_) and *D*_ν_(*T*_2_) are the single temperature spectra (STS), *B*_1,2_ are the coefficients which describe the contributions of each part.

The best fitting of the theoretical spectrum *Z*_ν_ to the experimental one *Y*_ν_ can be found by maximizing the correlation coefficient

(3) $$r=\frac{\sum Y_{\nu }\cdot Z_{\nu }}{\sqrt{\left(\sum Y_{\nu }^{2}\right)\cdot }\sqrt{\left(\sum Z_{\nu }^{2}\right)}}\to \max,$$

or by minimizing the residual sum of squares

(4) $$RSS=\sum \varepsilon_{\nu }^{2}=\sum {\left(Y_{\nu }-Z_{\nu }\right)}^{2}\to \min,$$

using a least square method (LSM) [17]. The minimum *RSS* can be expressed in the form.

(5) $$RSS_{\min}=\sum Y_{\nu }^{2}(1-r_{\max}^{2}).$$

This equation proves that both approaches—correlation technique and LSM—are identical.

## 4. Numeric Experiment

The proposed approach was first tested in a numerical experiment. At first the base *D*_ν_(*T_k_*) of the STS was constructed using the spectroscopic database HITRAN2016 for a temperature interval 300–2500 K with a temperature increment 10 K. A total pressure of 1 atm and a partial pressure of water vapor of 0.01 atm were presumed.

An arbitrary theoretical spectrum *D*_ν_(*T_j_*) for an arbitrarily selected temperature *T_j_* was then simulated and 100 random realizations of white noise with standard deviation *std* = 10^−3^ a.u. (absorbance unit) was added to *D*_ν_. By using a correlation technique, the best fit of this “noisy” simulated spectrum with the database spectra was found. This best fit provided the “unknown” temperature. This found temperature coincided with the initially preset one with an error Δ*T* about 10 K, which is mostly defined by the temperature grid of the STS base. This result demonstrates the efficiency of the correlation technique.

In the next step, a simulated spectrum was presented as the linear combination of the STS for two paths of equal length *L*, 1 atm pressure and with arbitrary temperatures *T*_1_ and *T*_2_. In these numerical experiments 100 random realizations of white noise with *std* = 5 × 10^−4^ a.u. were added to the combination of two STS. Thus constructed “noisy” spectrum was further treated as an experimental spectrum *Y_exp_*.

Initially, the spectrum *Y_exp_* was directly correlated with the base of STS. The maximum correlation coefficient provides *Т_eff_* which is actually the “mean” temperature on the assumption of a uniform LOS.

Then the constructed “experimental” spectrum *Y_exp_* was correlated with the sums of STS spectra with two different temperatures *T*_1_ and *T*_2_ with coefficients *B*_1_, and *B*_2_. The maximum correlation coefficient provided the estimation of maximum and minimum temperatures *T_high_* and *T_low_*. The estimated temperatures of the single realizations of *Y_exp_* were then averaged.

Some results of the numerical experiments are presented in Table 1. In the numerical experiments both temperatures in the different zones were postulated. The data presented in Table 1 were calculated for two low temperatures (500 and 700 K) while high temperatures varied from 900 K to 2000 K. Temperatures *Т_eff_* were calculated using the STS base with the assumption of a uniform LOS. Temperatures *T_high_*, *T_low_* were calculated using linear combinations of STS.

All values—*Т_eff_*, *T_high_* and *T_low_* were obtained from a maximum correlation coefficient. The sample estimation of a temperature evaluation was calculated using known expression

(6) $$\Delta T=\sqrt{\frac{\sum_{i=1}^{n}{\left(T_{i}-\bar{T}\right)}^{2}}{n-1}},$$

where $\bar{T}$ is the mean value of *n* realizations. The values Δ*T* were 20 K and 50 K for *T_low_* = 500 K and for *T_low_* = 700 K, respectively. The precision over 100 realizations of a simulated spectrum with randomly added white noise for the values *T_high_* presented in Table 1 was ~2 K for *T_low_* = 500 K and ~5 K for *T_low_* = 700 K.

The values *T_high_* coincide well with the introduced values *T*_2_ while *T_eff_* lies reasonably between *T_high_* and *T_low_*. It is noteworthy that the correlation coefficients for *T_eff_* are lower than for *T_high_* which is also understandable.

Finally, the LOS was modeled by a trapezium-form profile with a segment with a maximum temperature and a segment with linear decrease of the temperature from maximum to minimum. In these calculations the length of the section with a linear temperature gradient was 20% of the total length of the LOS. For this LOS profile, the *T_high_*, *T_low_* and *T_eff_* were found using the same correlation technique with linear combination of two STS. In these calculations *T_eff_* is again a “mean” temperature for the whole LOS. For the trapezium profile *T_low_* is the “mean” temperature of the segment with the temperature gradient. These results are shown in Table 2. The last numeric experiments showed that the preset *T_max_* for trapezium profile coincides with *T_high_* found above for two-temperature profiles of the LOS with an error of ~25 K. The results in Table 2 demonstrate the accuracy of this correlation technique for estimation of the maximum temperature in a non-uniform LOS.

## 5. Experimental

### 5.1. Experimental Setup

**Figure 2:**
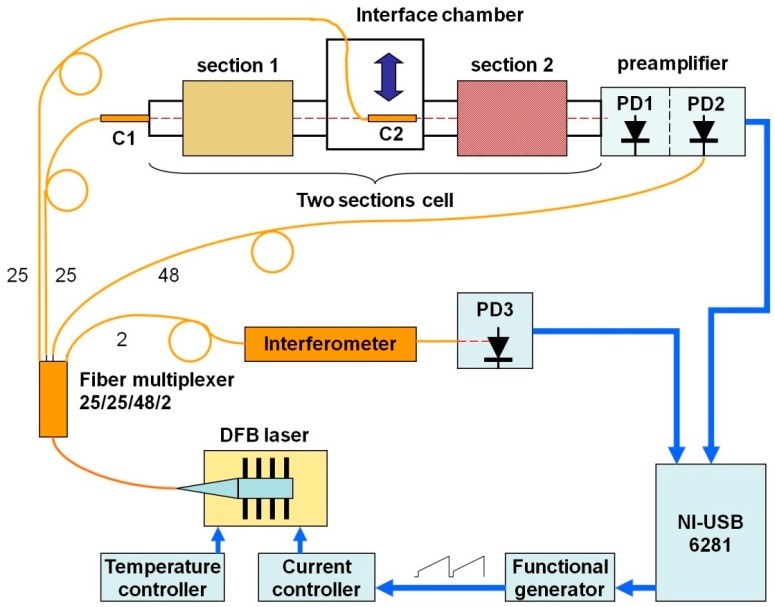
Experimental setup. C1 and C2 are collimators; PD1, PD2 and PD3 are photodetectors for the measurement, reference and wavenumber linearization channels, respectively.

Experimental setup is presented in Figure 2. A pigtailed distributed feedback diode laser (DFB DL Sacher Lasertechnik [18]) with output in the 1.343 μm (7460 cm^−1^) range was used in these experiments. The DL temperature and injection current controllers were TED350 and LDC202 (Thorlabs, Inc., Newton, NJ, USA, [19]), respectively. Injection current was additionally modulated by a trapeze waveform signal generated by a homemade generator. Due to this additional modulation, the DL wavelength could be regulated according to a preset program. During one scan the DL was tuned within an interval 7460–7463 cm^−1^. The repetition rate of DL scans was 250 Hz. Several absorption lines of H_2_O are located in this interval.

The output of the DL was divided into four portions by an optical fiber multiplexer in proportion 25:25:48:2. The minor part was delivered to the optical fiber interferometer and photodiode PD3 for relative wavelength scale calibration and linearization. Absolute calibration of the wavenumber scale was performed based on HITRAN2016. The largest part of DL output was delivered to the photodiode PD2 of the reference channel in the preamplifier. Two other parts were formed by the gradient collimators (C_1_, C_2_) and directed to the experimental cell. Depending on the mode of measurement the collimator C_2_ could be reproducibly inserted or removed from the optical axis. The transmitted beams were detected by the photodiode PD1. At the output of the preamplifier, differential signal and a signal proportional to the DL intensity were formed. The latter was used for signal normalization and measurement of the absolute absorbance. The output signals of the measurement and linearization channels were detected and processed by the Digital acquisition (DAQ) system based on the NI USB-6281 multifunction board (National Instruments, Austin, TX, USA [20]. The same board synchronized the function generator.

### 5.2. Cell

**Figure 3:**
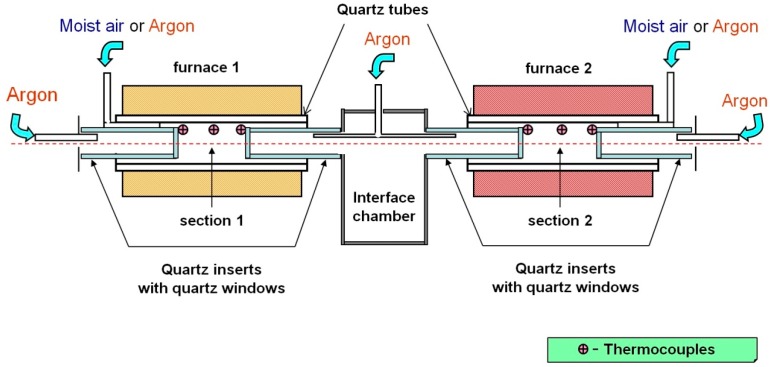
Two-section optical cell. The absorbing sections are located between inner windows of quartz inserts. Both sections can be separately heated by two independent electric furnaces. The inserts were flushed with dry argon to minimize the absorption of the diode laser (DL) beam outside the sections.

The scheme of the experimental cell is shown in Figure 3. The cell was combined from the equal parts. Two absorbing sections were formed between quartz windows of the two inserts. Temperature and water vapor concentrations in each section could be varied and controlled independently. Each section was located inside a quartz tube 30 cm long and 50 mm i.d. The tubes were located inside electrically heated cylindrical furnaces. The inserts were made of quartz tubes 30 cm long and 40 mm o.d. with quartz windows fixed at the inner sides of the inserts. The absorbing sections (space between the windows of the inserts) were 5 cm long. The temperature in each section was controlled by three commercial thermocouples PS2007 (Instrument Specialists Inc., Boerne, TX, USA). The accuracy of the thermocouples was 5 °C at 1000 °C. The ratio of the lengths of the sections (5 cm) to the lengths of the electrically heated tubes (30 cm) provided practically constant temperature of the absorbing gas in the sections.

The cylindrical inserts were continuously flushed with a stream of pure argon. The absorbing sections were filled either by pure argon or by air with regulated concentrations of water vapor. Both sections were fixed in line on a translation stage and connected by an interface chamber.

### 5.3. Measurement Procedure

For detection of the absorption spectra in uniform LOS the collimator C2 was inserted in the chamber (see Figure 2). The probing DL beam passed through Section 2 and was detected by the photodiode PD1. The registered signal was proportional to the absorbance in Section 2. Initially, the section was filled with dry argon and the measured signal was further used as the baseline (BL). Then the section was filled with moist air at 1 atm pressure. The temperature of the section was varied step-wise in the interval 700–1200 K with an increment of 50 K. The set of these absorption spectra registered for the whole range of temperatures were used to form the experimental STS_exp_ base.

For detection of the non-uniform absorption spectra the collimator C2 was removed from the optical axis. The DL probing beam formed by the collimator C1 passed through both sections. In this geometry the signal was proportional to the absorbance in two sections. Initially, the BL was measured with the two sections filled with dry argon. Then both sections were filled with moist air at 1 atm pressure. The temperature in Section 1 (*T*_1_) varied from 500 to 700 K while the temperature in Section 2 (*T*_2_) varied from 800 to 1200 K. At each measurement cycle the temperature of Section 1 was constant, the temperature of Section 2 was step-wise changed with an increment of 50 K. For each combination (*T*_1_, *T*_2_*)* 780 scans of the DL were registered for improving the signal-to-noise ratio. The absorption spectra measured for different (*T*_1_*, T*_2_*)* were further processed using the correlation technique.

## 6. Results and Discussion

**Figure 4:**
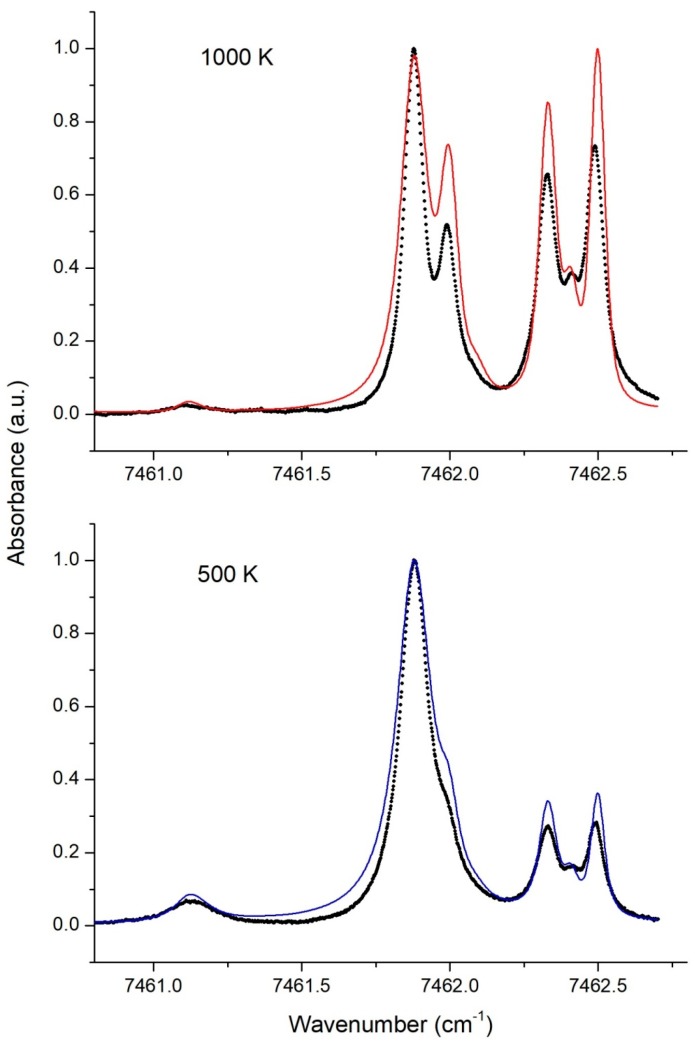
Simulated and experimental (dotted) spectra for two different temperatures. The spectra are normalized to the amplitude of the line at 7461.88 cm^−1^.

Experimental single temperature spectra under uniform conditions were recorded for different temperatures. In Figure 4 examples of the experimental (dots) and simulated spectra for two temperatures −500 K (blue line) and 1000 K (red line)—are presented. Simulated spectra were constructed using the HITRAN2016 base. Differences between the experimental and simulated spectral profiles are evident.

We believe that the main reason for such discrepancies is the lack of precise data on the width of the individual lines defined by collision broadening. This problem has already been pointed out in many publications [5,16,21,22,23,24].

**Figure 5:**
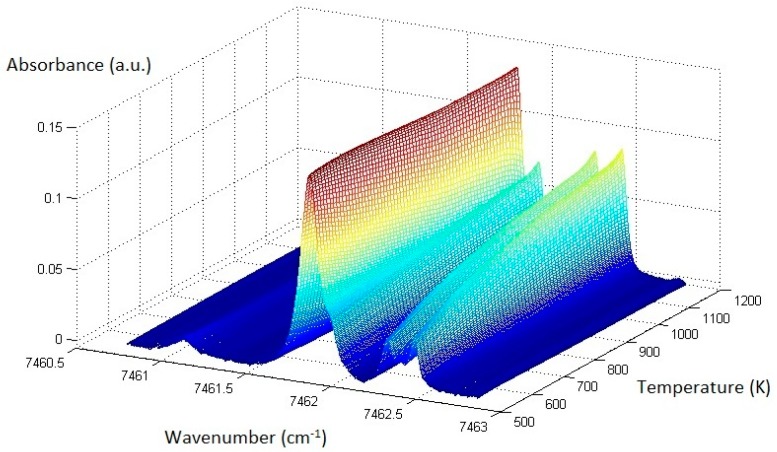
Experimental single temperature spectra (STS)_exp_ base.

The observed difference in temperature dependences stimulated the creation of our own experimental database. The procedure of the STS_exp_ construction is described above in Section 5.3. For numerical processing of the experimental spectra, a more detailed STS_exp_ base with a 10 K increment was constructed by linear interpolation of the 50 K grid. The structure of the resulting base with the 10 K increment is shown in Figure 5.

**Figure 6:**
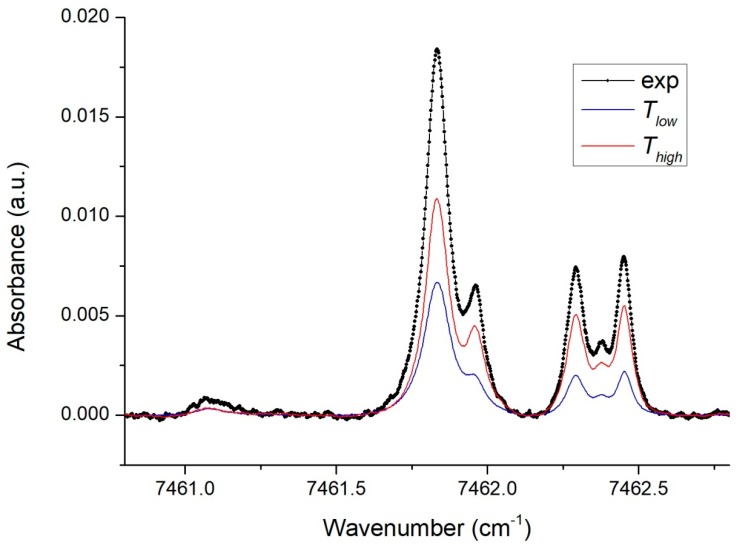
Experimental absorption spectrum of the two-section LOS (dots) and STS components the sum of which provides the best fit of the experimental spectrum: the high temperature STS is shown in red and the low temperature STS is shown in blue.

The experimental spectra were recorded for different combinations of high and low temperatures in two sections. The high temperature in Section 2 varied in the range 800–1200 K, the low temperature in Section 1 varied in the range 500–700 K. Each spectrum was processed by the proposed correlation algorithm using the created experimental STS_exp_ base. An example of the experimental spectrum for two specific temperatures in two sections is shown in Figure 6. In this example the actual temperatures in the sections measured by the thermocouples were *T*_1_ = 505 K and *T*_2_ = 921 K.

The experimental spectrum, shown dotted, was correlated with the experimental base STS_exp_. The STS components which provided the maximum correlation coefficient are marked in blue (low STS) and red (high STS). The best correlation was obtained in this example for *T_low_* = 600 K and *T_high_* = 890 K.

Using the described algorithm, high and low temperatures were estimated from the experimental spectra for different combinations of the conditions in both sections. The results are listed in Table 3.

In this Table the second and third columns show the temperatures measured in both sections by the thermocouples. Values of *T_eff_* calculated with the assumption of a uniform temperature distribution along the LOS are presented in the fourth column. Maximum correlation coefficients which defined *T_eff_* are listed in the fifth column. In the next two columns *T_low_* and *T_high_*, calculated using the two-zone model, are presented. In the last column the maximum correlation coefficients which provide the estimated *T_low_* and *T_high_* are listed. We estimate the accuracy of the temperatures in columns 2 and 3, each measured by three thermocouples, at 5 K. The statistical precision of the inferred temperature *T_high_* in these experiments was about 30 K.

The good coincidence of the temperatures measured by the thermocouples and the inferred values of *T_high_* should be noted. These results demonstrate the efficiency of the proposed algorithm for estimation of the highest temperature in a non-uniform LOS.

At the same time one should point out worse accuracy of *T_low_* estimation. It is the result of absorption line selection in our experiments. The primary goal of the investigations was to improve *T_high_* estimation, which dictated the selection of the optimal “hot” lines. The temperature dependence of the selected “cold” lines was much weaker as compared to the dependence of the “hot” lines. Because of the weak temperature dependence of “cold” lines the correlation coefficients were less sensitive to the variation of the “cold” component of the linear combination of STS (Equation (2)).

We also point out that the correlation coefficients for estimation of *T_eff_* are systematically lower than the coefficients for estimation of two temperatures in a non-uniform LOS. This is expected because it is clear that a non-uniform LOS cannot be characterized by one temperature. The decrease of the correlation coefficient can be partly a measure of non-uniformity of a LOS path.

## 7. Conclusions

A new algorithm for the evaluation of the maximum temperature in a spatially non-uniform hot zone by absorption spectroscopy with a tunable diode laser is proposed and experimental confirmed. The algorithm is based on the presentation of a resulting absorption spectrum of water vapor of a non-uniform zone as the sum of two single-temperature spectra with the appropriate variable coefficients. The best fit of the experimental and simulated spectra is found by a correlation technique in which the experimental spectra are correlated with the sum of single-temperature spectra. As a noticeable difference in the temperature dependences of the simulated and experimental spectra was observed, a single-temperature base was constructed from the experimentally registered single-temperature spectra. For numerical and experimental investigations the spectral range 7460–7463 cm^−1^ was selected. Several “hot” and “cold” absorption lines of water vapor lie within about 2.5 cm^−1^ in this interval. A single DFB diode laser operating in this range was used in the experiments. The absorbing cell was constructed of two different sections with independently regulated temperature and water vapor concentration.

Numerical experiments proved the efficiency of inferring maximum temperature in a two-temperature zone. In the experiments, the temperature in a “hot” section of the cell varied from 500–1200 K, while the temperature in a “cold” section varied from 500–700 K. The difference of the maximum temperature evaluation using the algorithm developed and the temperature measured by the thermocouples was about 50 K for 1100 K. The proposed algorithm can be useful if only maximum temperature but not the exact temperature spatial profile is of primary interest.

Our approach is similar to a two-zone profile fitting model [9], because LOS is considered as two zones with two different temperatures. In [9] the integral intensities for strong resolved H_2_O absorption lines were experimentally determined. Fitting of the experimental spectra are performed by construction of the set of equations for integral intensities of the individual absorption lines. In real combustion systems the H_2_O lines overlap, which makes more difficult to construct the set of equations. The algorithm developed allows inference of high temperature on LOS by fitting of the unresolved spectral structures using the correlation technique.

One should point out the general problem of the probing of a gas medium by any spectroscopic techniques. In real engines or propulsions the precise composition of gas mixture components is not completely known. Besides, the information on the broadening coefficients of different colliding partners is not complete, especially for high temperatures. Our approach works in the case where any specific feature in a spectrum depends on the temperature. Then the correlation technique used allows temperature estimation. In this paper such a feature does exist in the 7461.7–7462.3 cm^−1^ spectral interval.

## Figures and Tables

**Table 1:** Some results of the numeric experiments with two-temperature line-of-sight (LOS).

Preset Temperatures		Calculated Temperatures and Correlation Coefficients				
*T*_2_ (K)	*T*_1_ (K)	*T_eff_* (K)	*r_max_*	*T_high_* (K)	*T_low_* (K)	*r_max_*
900	500	710	0.99976	899.9	500.2	0.999859
1200	500	800	0.999539	1200.7	500.3	0.999842
1500	500	820	0.999133	1501.1	500.1	0.999794
2000	500	760	0.998375	1999.8	500.3	0.999548
1200	700	890	0.999859	1206.3	700.5	0.999896
1500	700	920	0.999737	1501.8	700.3	0.999842

**Table 2:** Some results of the numeric experiments with the trapezium temperature profile of LOS.

Preset Temperatures		Calculated Temperatures and Correlation Coefficients				
*T_max_* (K)	*T_gradient_* (K)	*T_eff_* (K)	*r_max_*	*T_high_* (K)	*T_low_* (K)	*r_max_*
900	300–900	850	0.99974	886	425	0.99975
1200	300–1200	1090	0.99971	1163	464	0.99973

**Table 3:** Results of the experimental spectra processing of the two-section LOS.

No	Thermocouple Temperatures		Calculated Temperatures and Correlation Coefficients				
	*T*_2_ (K)	*T*_1_ (K)	*T_eff_* (K)	*r_max_*	*T_high_* (K)	*T_low_* (K)	*r_max_*
1	781	576	670	0.9970	780	580	0.9981
2	888	700	820	0.9866	890	660	0.9906
3	921	505	780	0.9978	890	600	0.9983
4	990	580	780	0.9985	990	550	0.9985
5	1021	690	780	0.9968	1020	550	0.9983
6	1094	510	890	0.9993	1150	600	0.9997
7	1152	515	840	0.9961	1160	530	0.9975
8	1175	520	930	0.9970	1180	580	0.9985

## References

[1] Allen M.G. (1998). Diode laser absorption sensors for gas-dynamic and combustion flows. Meas. Sci. Technol..

[2] Lackner M. (2007). Tunable Diode Laser Absorption Spectroscopy (TDLAS) in the Process Industries—A Review. Rev. Chem. Eng..

[3] Schulz C., Dreizler A., Ebert V., Wolfrum J., Tropea C., Yarin A.L., Foss J.F. (2007). Combustion Diagnostics. Springer Handbook of Experimental Fluid Mechanics.

[4] Bolshov M.A., Kuritsyn Y.A., Romanovskii Y.V. (2015). Tunable diode laser spectroscopy as a technique for combustion diagnostics. Spectrochim. Acta Part B At. Spectrosc..

[5] Goldenstein C.S., Spearrin R.M., Jeffries J.B., Hanson R.K. (2017). Infrared laser-absorption sensing for combustion gases. Prog. Energy Combust. Sci..

[6] Ouyang X., Varghese P.L. (1989). Line-of-sight absorption measurements of high temperature gases with thermal and concentration boundary layers. Appl. Opt..

[7] Sanders S.T., Wang J., Jeffries J.B., Hanson R.K. (2001). Diode-Laser Absorption Sensor for Line-of-Sight Gas Temperature Distributions. Appl. Opt..

[8] Palaghita T., Seitzman J.M. (2006). Absorption-based temperature-distribution-sensing for combustor diagnostics and control. Proceedings of the 44th AIAA Aerospace Sciences Meeting and Exhibit.

[9] Liu X., Jeffries J.B., Hanson R.K. (2007). Measurement of Nonuniform Temperature Distributions Using Line-of-Sight Absorption Spectroscopy. AIAA J..

[10] Gordon I.E., Rothman L.S., Hill C., Kochanov R.V., Tan Y., Bernath P.F., Birk M., Boudon V., Campargue A., Chance K.V. (2017). The HITRAN2016 molecular spectroscopic database. J. Quant. Spectrosc. Radiat. Transf..

[11] Rothman L.S., Gordon I.E., Barber R.J., Dothe H., Gamache R.R., Goldman A., Perevalov V.I., Tashkun S.A., Tennyson J. (2010). HITEMP, the high-temperature molecular spectroscopic database. J. Quant. Spectrosc. Radiat. Transf..

[12] Wagner S., Klein M., Kathrotia T., Riedel U., Kissel T., Dreizler A., Ebert V. (2012). In situ TDLAS measurement of absolute acetylene concentration profiles in a non-premixed laminar counter-flow flame. Appl. Phys. B.

[13] Ma L., Li X., Sanders S.T., Caswell A.W., Roy S., Plemmons D.H., Gord J.R. (2013). 50-kHz-rate 2D imaging of temperature and H_2_O concentration at the exhaust plane of a J85 engine using hyperspectral tomography. Opt. Express.

[14] Cai W., Kaminski C.F. (2017). Tomographic absorption spectroscopy for the study of gas dynamics and reactive flows. Prog. Energy Combust. Sci..

[15] Liu X. (2006). Line-of-Sight Absorption of H_2_O Vapor: Gas Temperature Sensing in Uniform and Nonuniform Flows. Ph.D. Thesis.

[16] Liger V.V., Kuritsyn Y.A., Mironenko V.R., Bolshov M.A., Ponurovskii Y.Y., Kolesnikov O.M. (2018). Measurement of Non-Stationary Gas Flow Parameters Using Diode Laser Absorption Spectroscopy at High Temperatures and Pressures. High Temp..

[17] Least-Squares (Model Fitting) Algorithms MathWorks. http://www.mathworks.com/help/optim/ug/least-squares-model-fitting-algorithms.html.

[18] Sacher Lasertechnik. http://www.sacher-laser.com/home/laser-diodes/distributed_feedback_laser/dfb/single_mode.html.

[19] Thorlabs, Inc.. https://www.thorlabs.com/index.cfm.

[20] NI USB-6281. http://www.ni.com/en-us/shop/select/multifunction-io-device?modelId=124928.

[21] Liu X., Jeffries J.B., Hanson R.K. (2007). Measurements of spectral parameters of water-vapour transitions near 1388 and 1345 nm for accurate simulation of high-pressure absorption spectra. Meas. Sci. Technol..

[22] Liu X., Zhou X., Jeffries J.B., Hanson R.K. (2007). Experimental study of H_2_O spectroscopic parameters in the near-IR (6940–7440cm^−1^) for gas sensing applications at elevated temperature. J. Quant. Spectrosc. Radiat. Transf..

[23] Wang Q., Chang J., Kong D.L., Liu Y.N., Wang F.P., Zhu C.G., Wei W., Liu X.Z. (2014). Optical Measurement of Water Vapor Concentration and Gas Pressure. IEEE Sens. J..

[24] Schroeder P.J., Pfotenhauer D.J., Yang J., Giorgetta F.R., Swann W.C., Coddington I., Newbury N.R., Rieker G.B. (2017). High temperature comparison of the HITRAN2012 and HITEMP2010 water vapor absorption databases to frequency comb measurements. J. Quant. Spectrosc. Radiat. Transf..

